# Methods for rapid frequency-domain characterization of leakage currents in silicon nanowire-based field-effect transistors

**DOI:** 10.3762/bjnano.5.110

**Published:** 2014-07-04

**Authors:** Tomi Roinila, Xiao Yu, Jarmo Verho, Tie Li, Pasi Kallio, Matti Vilkko, Anran Gao, Yuelin Wang

**Affiliations:** 1 Department of Automation Science and Engineering, Tampere University of Technology, Tampere, Finland; 2 Shanghai Institute of Microsystem and Information Technology, Chinese Academy of Sciences, Shanghai, China

**Keywords:** admittance spectroscopy, excitation design, frequency characterization, frequency response, silicon nanowire

## Abstract

Silicon nanowire-based field-effect transistors (SiNW FETs) have demonstrated the ability of ultrasensitive detection of a wide range of biological and chemical targets. The detection is based on the variation of the conductance of a nanowire channel, which is caused by the target substance. This is seen in the voltage–current behavior between the drain and source. Some current, known as leakage current, flows between the gate and drain, and affects the current between the drain and source. Studies have shown that leakage current is frequency dependent. Measurements of such frequency characteristics can provide valuable tools in validating the functionality of the used transistor. The measurements can also be an advantage in developing new detection technologies utilizing SiNW FETs. The frequency-domain responses can be measured by using a commercial sine-sweep-based network analyzer. However, because the analyzer takes a long time, it effectively prevents the development of most practical applications. Another problem with the method is that in order to produce sinusoids the signal generator has to cope with a large number of signal levels. This may become challenging in developing low-cost applications. This paper presents fast, cost-effective frequency-domain methods with which to obtain the responses within seconds. The inverse-repeat binary sequence (IRS) is applied and the admittance spectroscopy between the drain and source is computed through Fourier methods. The methods is verified by experimental measurements from an n-type SiNW FET.

## Introduction

Recent development in sensing biochemical molecules has been rapid. Among many sensing technologies, silicon nanowire (SiNW)-based field-effect transistors (FETs) [1] are one of the most promising building blocks for the next generation of electrical circuits in recognizing a wide range of biological and chemical targets. They have been successfully used in the detection of DNA [2], pH [3], protein [4], glucose [5], virus [6], and vapor [7]. Despite significant developments, underlying detection mechanism and dynamics of the SiNW FETs are not well-defined, and hence, further studies and methods are required [8].

**Figure 1:**
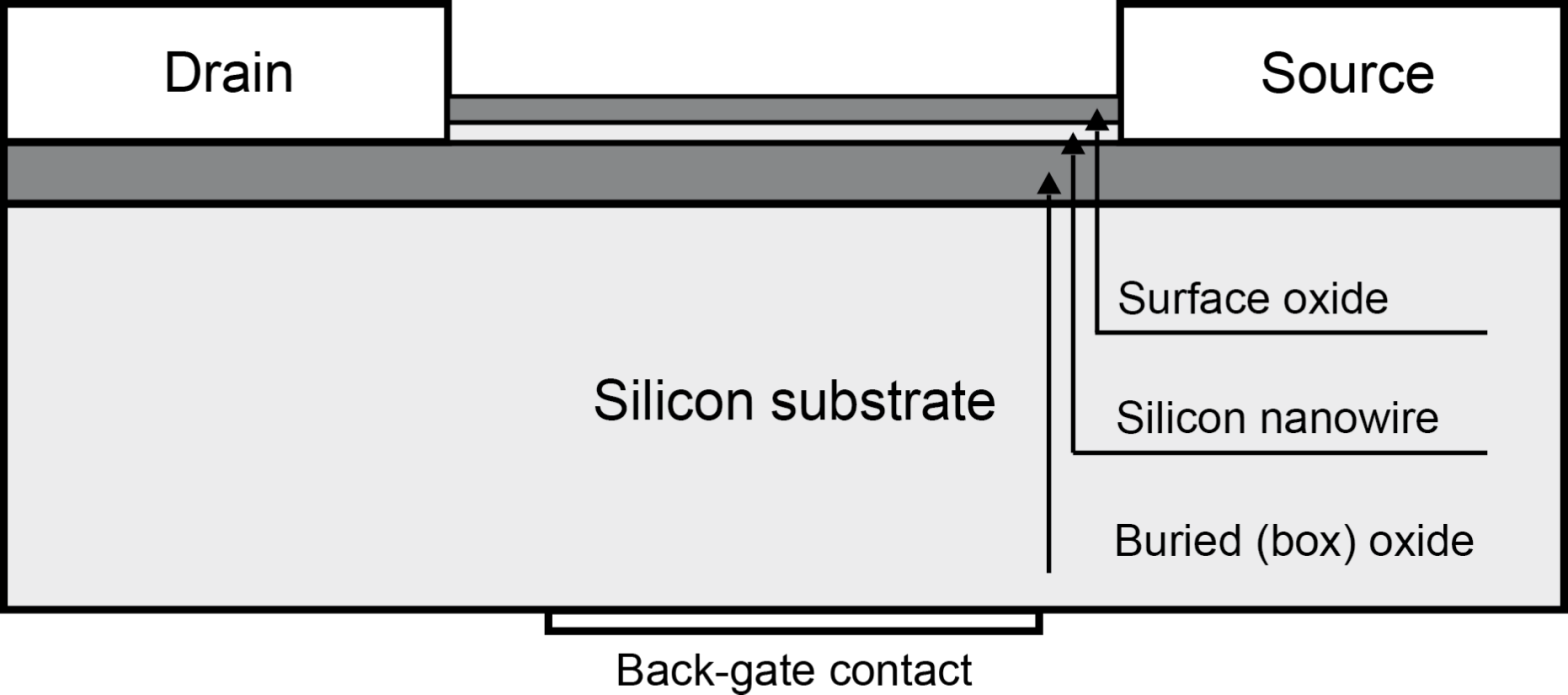
Conceptual diagram of a SiNW FET.

Due to the large surface-to-volume ratio, one-dimensional nanostructures are one of the best candidates for ultra-sensitive sensors. The most typical configuration applies a nanowire as the essential building block, bonding two ends of the nanowire to a solid substrate to create a SiNW FET. Figure 1 shows the structure of a typical SiNW FET. The thin SiNW body is electrically isolated from the silicon substrate by a buried oxide (box) layer. Back-gate contact is used to control the conductivity of the SiNW with the box layer. When applying a threshold voltage on the silicon substrate, SiNW conductivity varies rapidly with small changes in the potential of the nanowire surface, which are induced by the molecules (detection targets) adsorbed on the surface oxide of the nanowire. The conductivity change is seen as a change in the voltage–current behavior (*V*_ds_–*I*_ds_ behavior) between the drain and source. Some current, known as leakage current, flows between the gate and drain of the transistor, and affects *I*_ds_. Recent studies by the authors indicate that the leakage current is likely to be frequency-dependent. Accurate measurements of such frequency-dependent characteristics can be used to validate the functionality of the SiNW FETs and to determine their most effective operation ranges. In addition, the measurements could possibly be advantageous in developing new detection technologies utilizing SiNW FETs.

Based on an extensive literature review, the leakage current has not been previously measured or characterized in the frequency domain, with the exception of the work of the authors. The number of studies considering frequency-domain measurements of SiNW FETs is very low. The authors in [9] applied low-frequency noise spectroscopy (LFNS), and characterized generation–recombination centers in silicon nanowires grown by using chemical vapor deposition. Their aim was to demonstrate the potential of the LFNS in characterizing deep levels in nanowires. The authors in [10] presented a novel protein-detection methodology based on frequency-domain electrical measurements. They demonstrated that the power spectral density of voltage from a current-biased SiNW FET showed 1/*f*-dependence in the frequency domain in the presence of protein not specific to the antibody receptor. However, in the presence of protein recognized by the SiNW FET, the spectral density of the voltage exhibited a Lorentzian shape with a frequency of several kilohertz. The authors in [11] demonstrated by simulations that nanowires exhibited an AC-transfer function that resembled that of a high-pass filter. They showed that the corner frequency of the filter decreases as more molecules, corresponding to a higher net charge, attached to the nanowire and displaced more charge carriers in the nanowire channel. The results provided the means to build a low-cost frequency-based detection system when applying the SiNW FET.

Despite the impressive results of past studies, frequency-domain analysis of the SiNW FETs has not gained much popularity. One of the main reasons is that efficient frequency-response-measurement techniques are not well known. All the measurements in these studies were performed by using a sine-sweep-based network analyzer. Although the method usually yields reliable and accurate responses, it suffers from a number of drawbacks. The most important of these is the length of time required for the measurement. Responses are measured separately at various frequencies by applying single sinusoids. Consequently, one measurement cycle usually takes several minutes, which makes the method inefficient for most practical applications. Another problem with the method is that in order to produce sinusoids the signal generator has to cope with a large number of signal levels. This may become challenging in developing low-cost applications. Instead of using single sinusoids, an excitation signal with a broadband spectrum can be generated to gather all the spectral information in one measurement. There exists a multitude of such signals. The authors in [12] listed ten different signals in their survey. One special class of these signals is the maximum-length binary sequence (MLBS). The sequence is one class of periodic pseudo-random multi-frequency signals. The MLBS has energy at several frequencies. This makes it possible to simultaneously measure a frequency response at those frequencies through Fourier methods [13]. Therefore, instead of measuring a response separately at each frequency, all the required information can be captured within one measurement. This drastically reduces the measurement time from several minutes to few seconds. The short measurement time has many advantages in the presented application. For example, the possible heating effect during the measurement procedure can be minimized. In addition, the fast measurement technique has significant advantages over the slow method for practical applications such as detecting chemical targets.

In this paper, the inverse-repeat binary sequence (IRS) is applied as an excitation injection. The IRS is used to characterize linear dynamics from nonlinear systems [14]. The sequence is generated from the conventional MLBS by doubling the MLBS and toggling every other bit of the doubled sequence. Because the sequence has a binary form, it can be easily generated by a low-cost application. The efficiency of the IRS excitation has been demonstrated in numerous applications, such as chemical process systems [15], analysis of a reservoir pipeline-valve system [16], and in frequency-response measurement of switched-mode power supplies [17].

This paper presents fast frequency-domain methods with which to measure and characterize the leakage current in SiNW FETs. The primary aim of the work is to present a cost-effective implementation to obtain the leakage current in the frequency domain. The work is an extended and revised version of the results in [18]. The measurement technique is further improved by applying the IRS excitation instead of the MLBS. In addition, the applied device is described more in detail, and the measurement results are more comprehensive. The theoretical backgrounds of the methods are introduced, followed by the experimental results from an n-type SiNW FET.

## Methods

**Figure 2:**
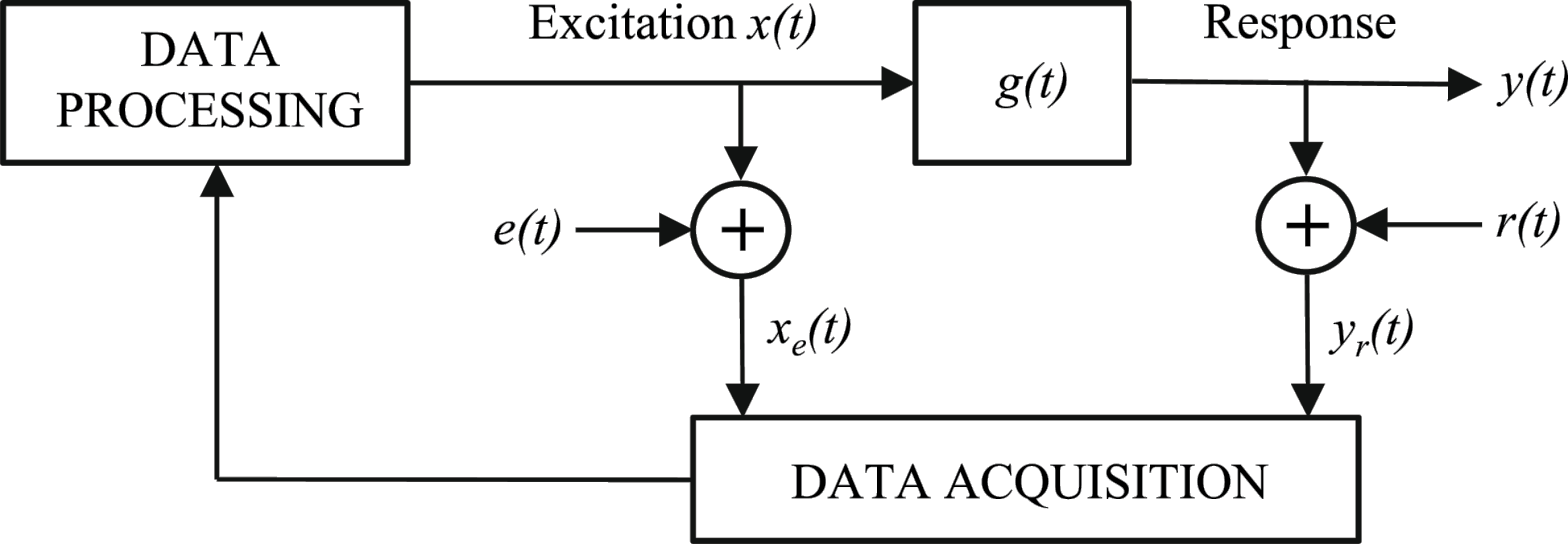
Typical measurement system.

Consider the system *g*(*t*) shown in Figure 2 as a linear time-invariant system for small disturbances. According to basic control theory, the system can be fully characterized by its impulse response(s), which can be transformed into frequency domain and presented by the frequency-response function (FRF) [19]. The excitation *x*(*t*) is injected into the system and the output response *y*(*t*) is obtained. The input and output noises (*e*(*t*) and *r*(*t*)) disturb the measurements that can be now denoted by *x_e_*(*t*) and *y_r_*(*t*).

The frequency-response function of the device can be denoted as

[1]
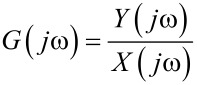


where *Y*(*jω*) and *X*(*jω*) denote the Fourier transforms of the corresponding time-domain signals *y*(*t*) and *x*(*t*). In the presence of external noise the noise-affected frequency-response function *G_n_*(*jω*) can be denoted as

[2]
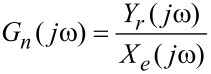


where *X_e_*(*jω*) and *Y_r_*(*jω*) denote the Fourier transforms of *x_e_*(*t*) and *y_r_*(*t*). Denoting the error signals *e*(*t*) and *r*(*t*) by their Fourier transforms *E*(*jω*) and *R*(*jω*), *G_n_*(*jω*) becomes

[3]
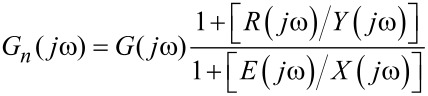


Clearly, in the presence of noise at the input and output signals, the measured transfer function obtained by Equation 3 may significantly differ from the real frequency-response function *G*(*jω*). To avoid the problem, the cross-correlation technique has been often applied [20]. In this method, a cross-correlation is computed between the perturbation and sensed output after which the frequency response is obtained by Fourier transform. The method tends to cancel out the effect of external noise in output provided that the excitation resembles ideal white noise. The method requires, however, that the measured perturbation is noise free. Considering a system shown in Figure 2, the ideal perturbation is not measured and used in the computation. Hence the cross-correlation technique fails and both random and bias error will be present. Even in the case of ideal perturbation, the cross-correlation technique reduces the effect of noise only from the output side. In the presence of noise both at input and output, the logarithmic averaging procedure [21] is proposed as

[4]
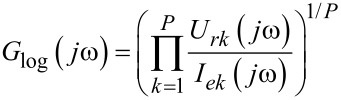


where *P* denotes the number of injected excitation periods. In the method, the measurements from both input and output sides are segmented and Fourier transformed after which Equation 4 is applied. As the conventional cross-correlation method requires that the excitation resembles ideal white noise (or more specifically, the auto correlation of the excitation resembles Dirac delta function), the logarithmic averaging procedure does not have this requirement. The method tends to cancel out the effect of uncorrelated noise both from input and output sides, and hence, the frequency response is obtained more accurately [21].

### Inverse-repeat binary sequence

The MLBS is an efficient excitation sequence for various frequency-domain analysis [22]. However, an assumption with MLBS is, that the process under consideration is linear. It may be obvious that practical transistors and related measurement systems are always affected by some nonlinearities, such as the quantization effect. The nonlinear phenomena should be minimized to increase the accuracy of the measurements. There are basically two methods to model systems that exhibit nonlinearities. The first and more difficult method is to identify a model that includes all the system nonlinearities. The second and more practical method is to identify only the linear part of the model. One the most convenient way to minimize the effect of nonlinearities is to carefully select the excitation injection.

**Figure 3:**
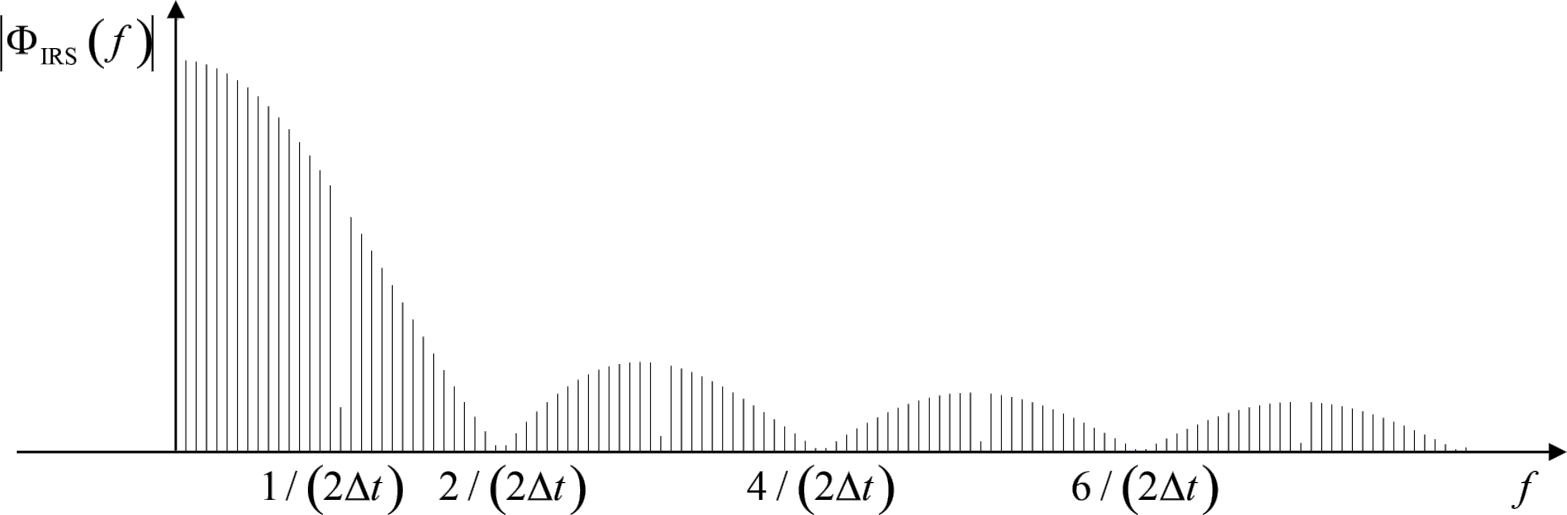
Shape of the power spectrum of IRS from 5-bit length shift register.

The inverse-repeat binary sequence (IRS) has been a popular excitation signal for nonlinear systems to characterize the linear part. The sequence is generated from the MLBS by doubling the MLBS and toggling every other bit of the doubled sequence. By using an adequately long signal length and a sufficiently short clock cycle, the spectral and auto-correlation properties of the IRS are very close to the corresponding properties of a pure white noise. The sequence has the lowest possible peak factor, which means that the signal energy is very high in relation to the signal amplitude [23]. Another advantage of the signal is that it can be generated with a low-cost application, the output of which can only cope with a small number of signal levels. Furthermore, leakage in the frequency-response calculation can be avoided because the sequence is periodic. The IRS is a highly acceptable alternative to be applied with (4) due to its capability to suppress the undesired effect of nonlinear phenomena. As an example, the power spectrum of the IRS generated by a five-bit length shift register [24] is shown in Figure 3. The power drops towards zero at half of the generation frequency of 1/2Δ*t* and its harmonics.

It should be emphasized that signal energy in the IRS is divided into a number of frequency harmonics, as shown in Figure 3. The FRF may become difficult to obtain under high signal-to-noise (SNR) requirements if the excitation amplitude cannot be increased due to system sensitivities. Furthermore, the IRS may not make significant improvements in the FRF measurements compared to the results of the MLBS in the case of a system with strong odd-order nonlinearities [17]. A straightforward IRS design procedure is well documented. The authors in [17] considered the frequency-response measurements of switched-mode power supplies, and applied the IRS to analyze the output impedance. The design steps of the excitation sequence can also be applied for the application in this paper.

## Results and Discussion

**Figure 4:**
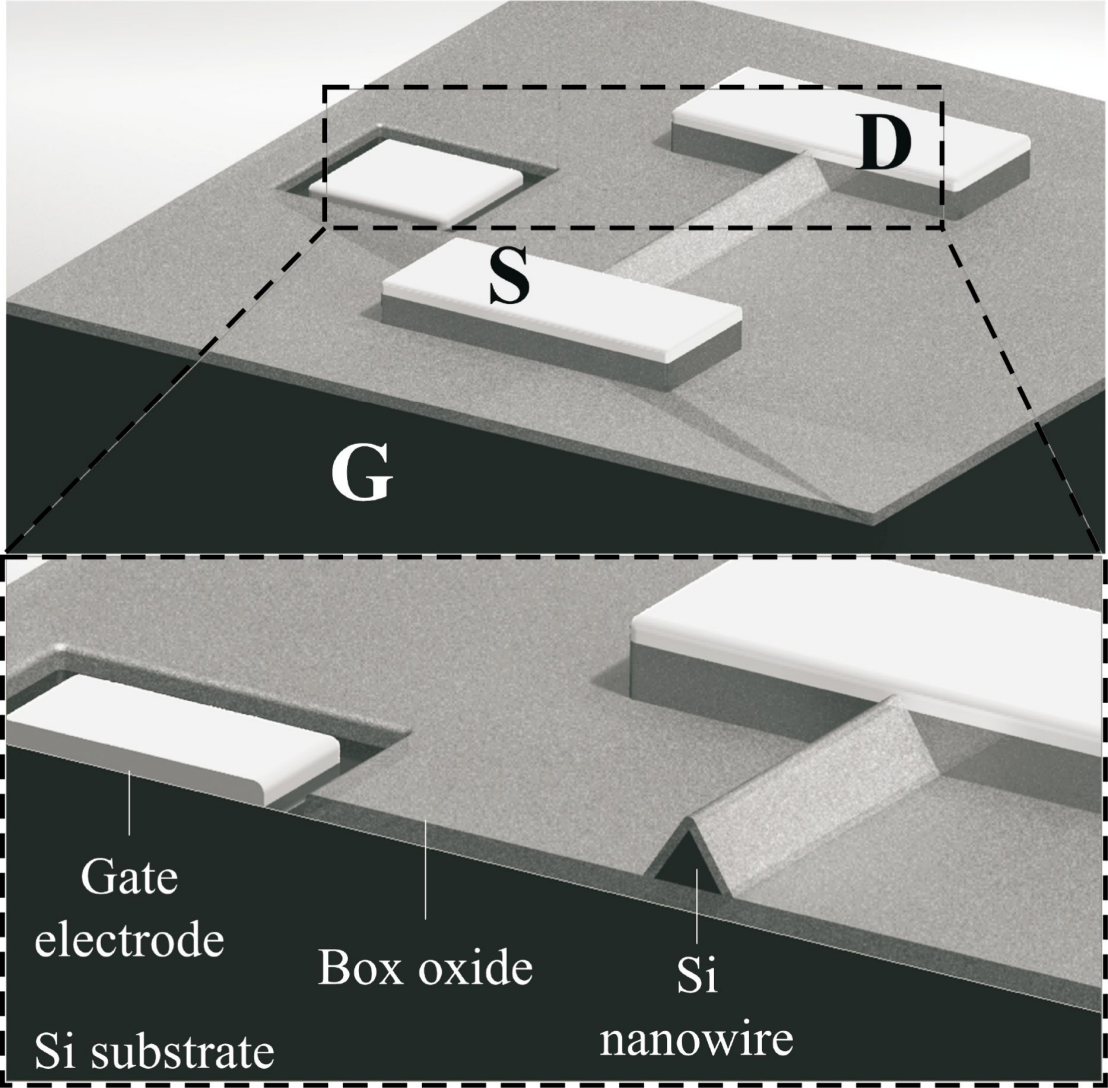
Schematic of the applied SiNW FET device.

The presented methods were applied, and the leakage current was characterized from a SiNW FET. An n-type SiNW FET device was fabricated by using a silicon-on-insulator (SOI) wafer with a top-down method. A full description of the fabrication process is given in [25]. The structure of the device is schematically shown in Figure 4, where G, S, and D denote the gate, source, and drain, respectively.

**Figure 5:**
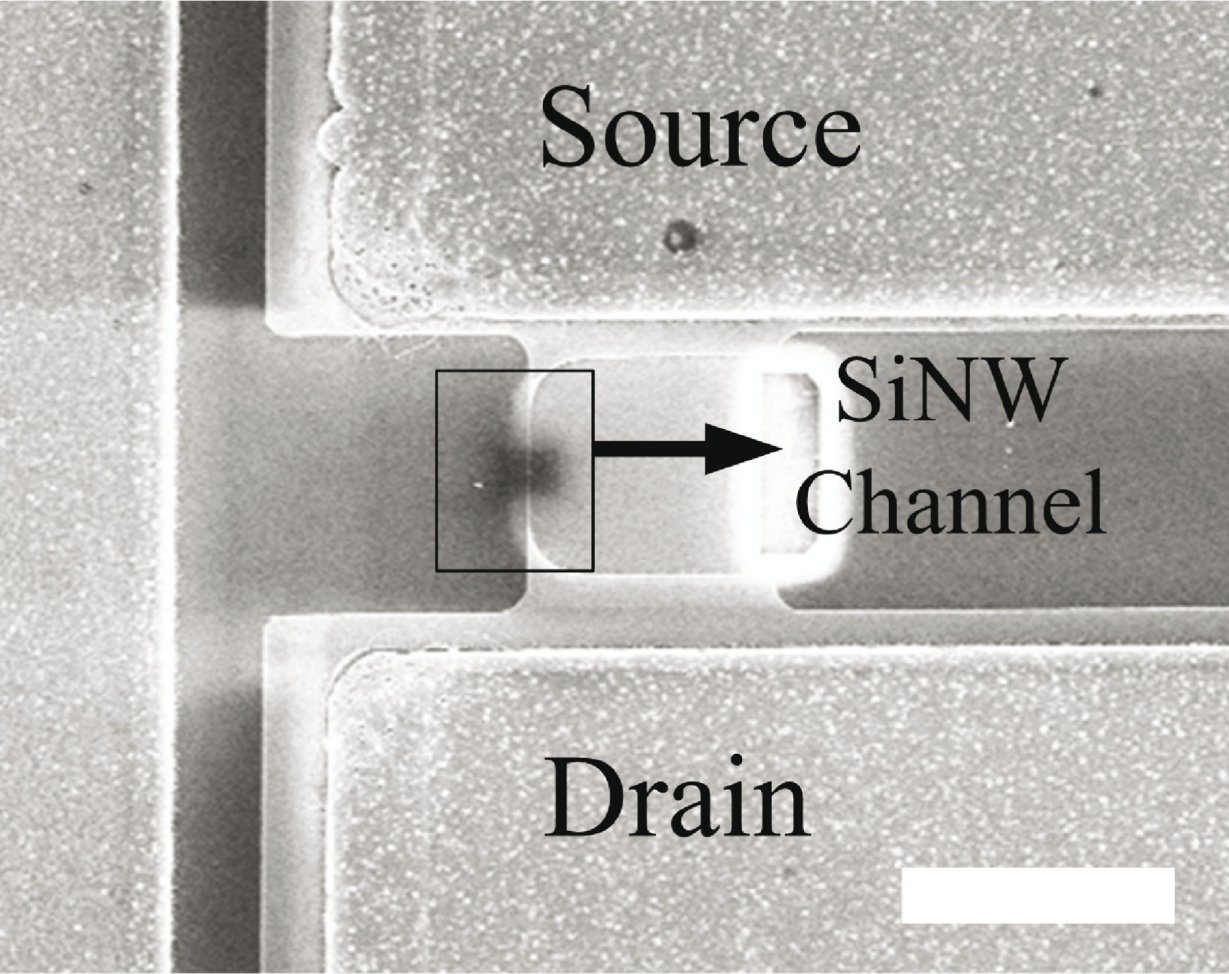
SEM image of the SiNW FET device. The scale bar is 20 µm.

**Figure 6:**
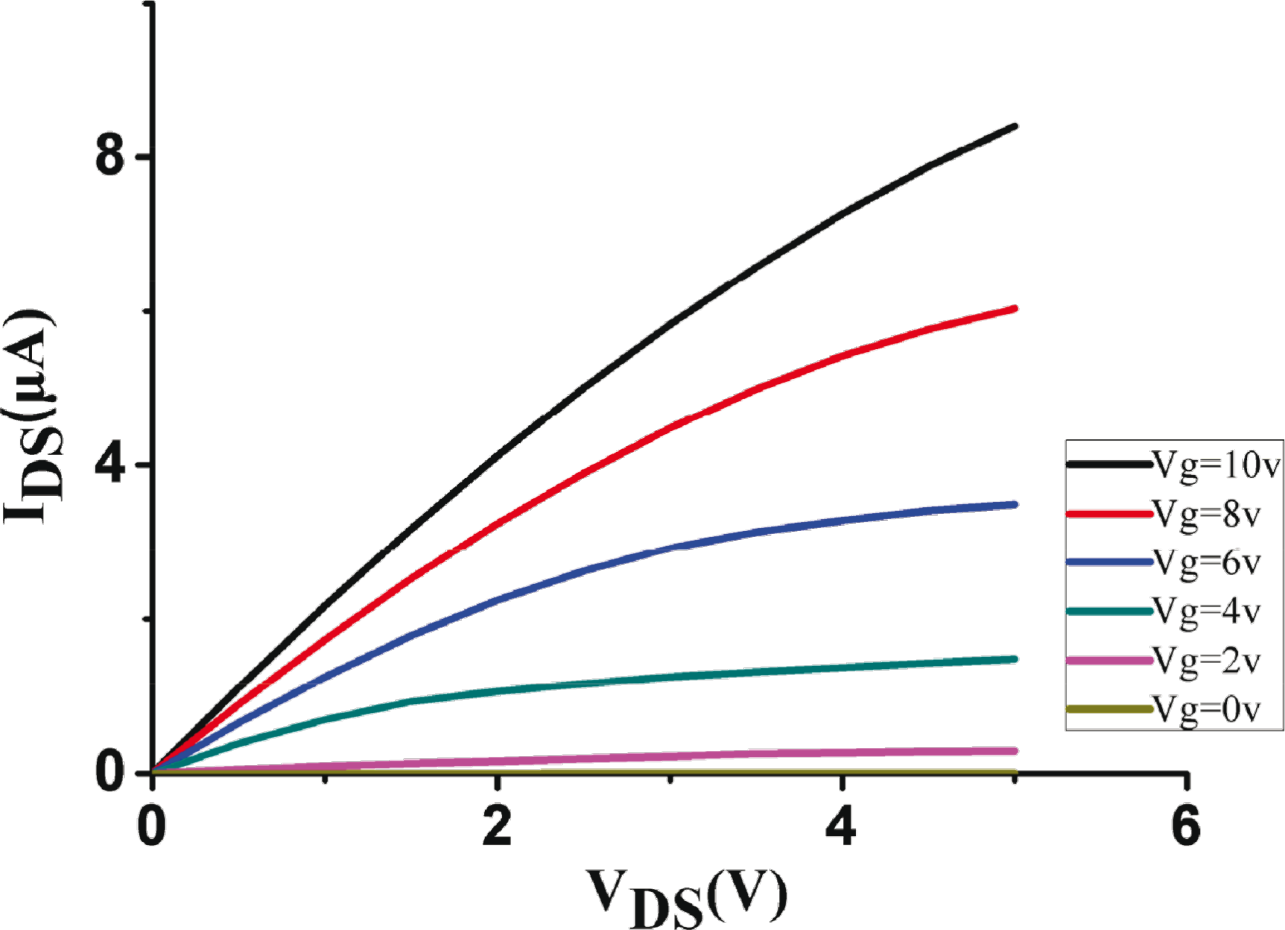
*I*_ds_–*V*_ds_ DC measurement results.

**Figure 7:**
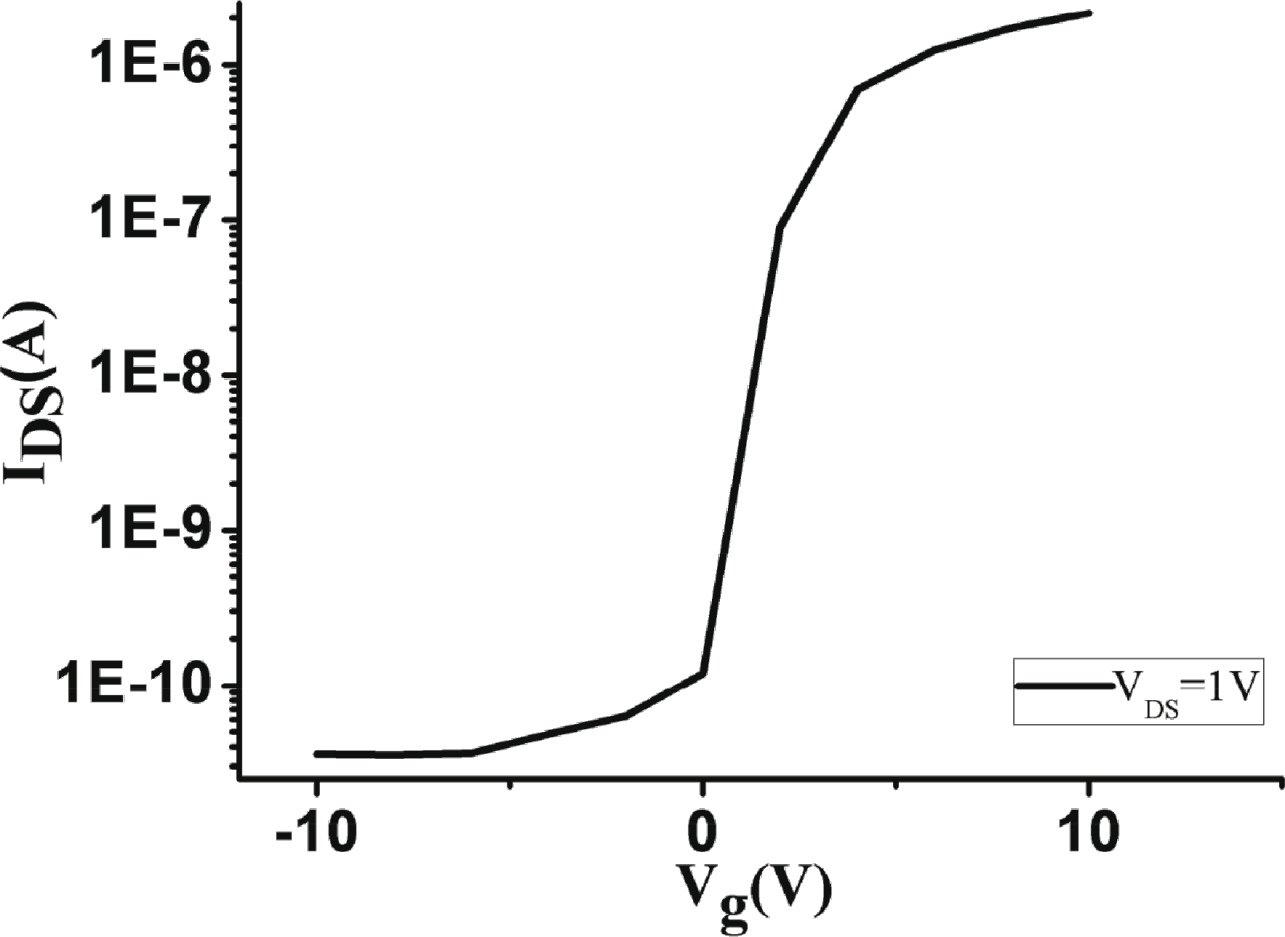
*I*_ds_–*V*_g_ DC measurement results.

A scanning electron microscope (SEM) image of the device is shown in Figure 5. The source and drain regions are heavily doped with boron, while the nanowire channel has a low-doping concentration of phosphorous. The contacts were doped by different dopants in order to form an off-state channel while there was no gate voltage applied on it thus saving energy. The cross-section of the SiNW is a triangle, with bottom width of 50 nm, and height of 65 nm. The length of the SiNW is 10 μm. The doping concentration is 3·10^15^/cm^3^. The DC measurements (*I*_ds_–*V*_ds_ and *I*_ds_–*V*_g_) of the device are shown in Figure 6 and Figure 7.

**Figure 8:**
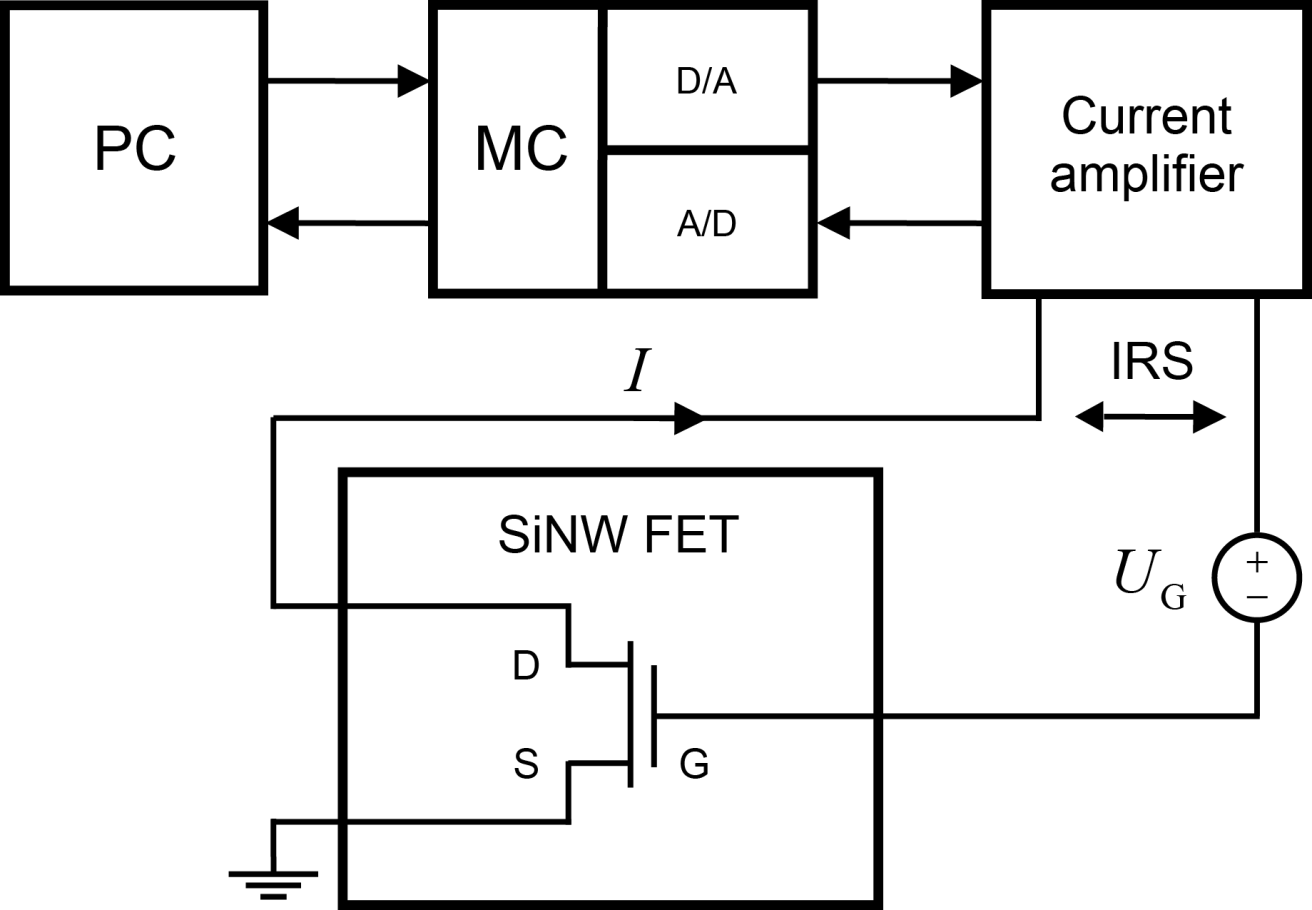
Conceptual diagram of the measurement setup.

Figure 8 shows the measurement setup. The gate voltage of the SiNW FET is controlled by *U*_G_. The interface board communicating between the computer and the device under test is National Instruments USB-6251 measurement card (MC). The maximum sampling frequency of the card is 1 MHz (aggregate), and the maximum digital-to-analog converting frequency (maximum generation frequency of the excitation) is 2.8 MHz. The measured data is digitized by a 16-bit analog-to-digital (A/D) converter. The measurement card is controlled by a Matlab/Data Acquisition toolbox.

**Figure 9:**
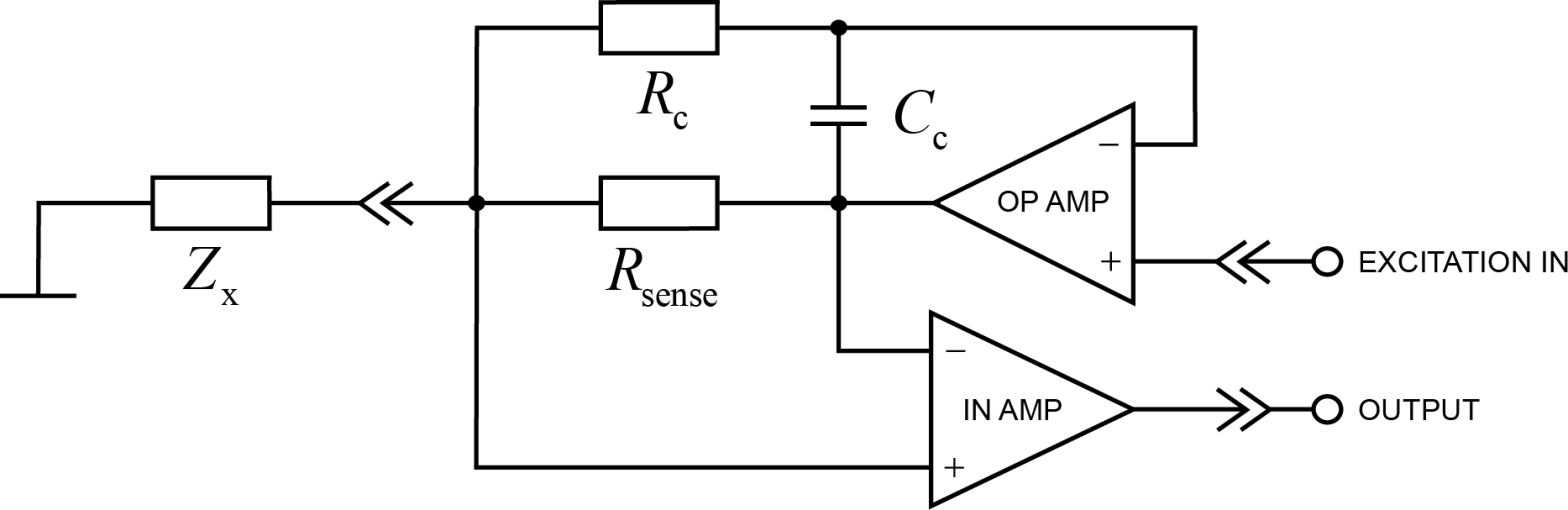
Simplified schematic of the measurement amplifier.

Because the measurement card was not directly suitable for measuring very small currents, an amplifier was designed to interface with the measurement setup. The amplifier can measure currents in the range of 1–1000 nA (at an excitation voltage level of ±1 mV). Figure 9 shows a simplified schematic of the amplifier. The upper operational amplifier in the schematic works as an excitation voltage buffer, which drives the ground-referred unknown impedance *Z*_x_ through a current sense resistor *R*_sense_. The measured current causes a voltage drop in this resistor, which is then amplified with the lower instrumentation amplifier. *C*_c_ and *R*_c_ are added to prevent oscillation in case *Z*_x_ is highly capacitive. Excitation voltage scaling, power supplies, filtering, and additional amplifier stages are not included. The amplifier has a selectable gain of −1.1 mV/nA or −4.5 mV/nA. The bandwidth depends on the impedance to be measured, and varies in the range from 10 to 100 kHz.

The IRS was synthesized by an eight-bit length shift register [24]. The measurable bandwidth was limited by the current amplifier, and was approximately 70 kHz. The generation frequency of the measurement card was set to 100 kHz to provide enough energy to the whole frequency band. Because the measured signal levels were extremely low, a large number of excitation periods and averaging were used to reduce the effect of noise. Two hundred periods were used in the experiment. The total length of the excitation signal was *L* = 200·2·(2^8^ − 1) = 102,000. With these parameters, the lowest frequency harmonic was 100 kHz/510 ≈ 196 Hz. The sampling frequency *f*_o_ was set to four times the generation frequency (400 kHz) to provide reasonable multi-sampling [17]. Table 1 summarizes the parameters used in the experiment.

**Table 1:** Parameters used in the experiment.

parameter	value	unit

excitation signal length, *P*	510	bits
generation frequency, *f*_gn_	100	kHz
number of excitation periods, *R*	200	
excitation amplitude, *A*	1	mV
sampling frequency, *f*_o_	400	kHz

**Figure 10:**
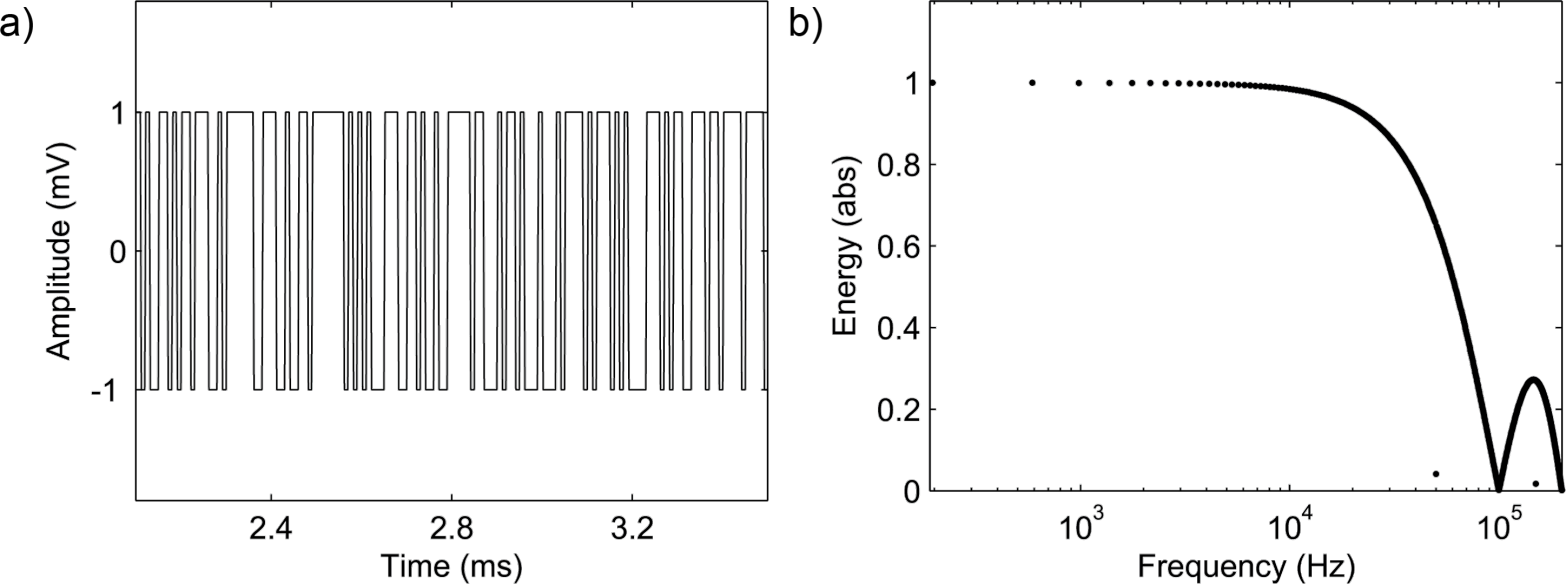
Generated excitation sequence; a) sample in the time domain, and b) (scaled) energy content.

The designed IRS was then injected into the SiNW FET through the current amplifier. Figure 10a shows a sample of the generated IRS in the time domain. Figure 10b shows the (scaled) power spectra. The voltage between the gate and drain was measured, together with the corresponding current. The device was assumed to maintain approximately the constant operating point during multiple excitations. The total amount of collected data was 408,000 samples. The process of collecting the data took 4*L*/*f*_o_ = 1.02 s. The collected data was divided into segments, each with a length of one full multi-sampled excitation period (4·510 = 2,040). The logarithmic averaging procedure (Equation 4) was then applied to each data period.

**Figure 11:**
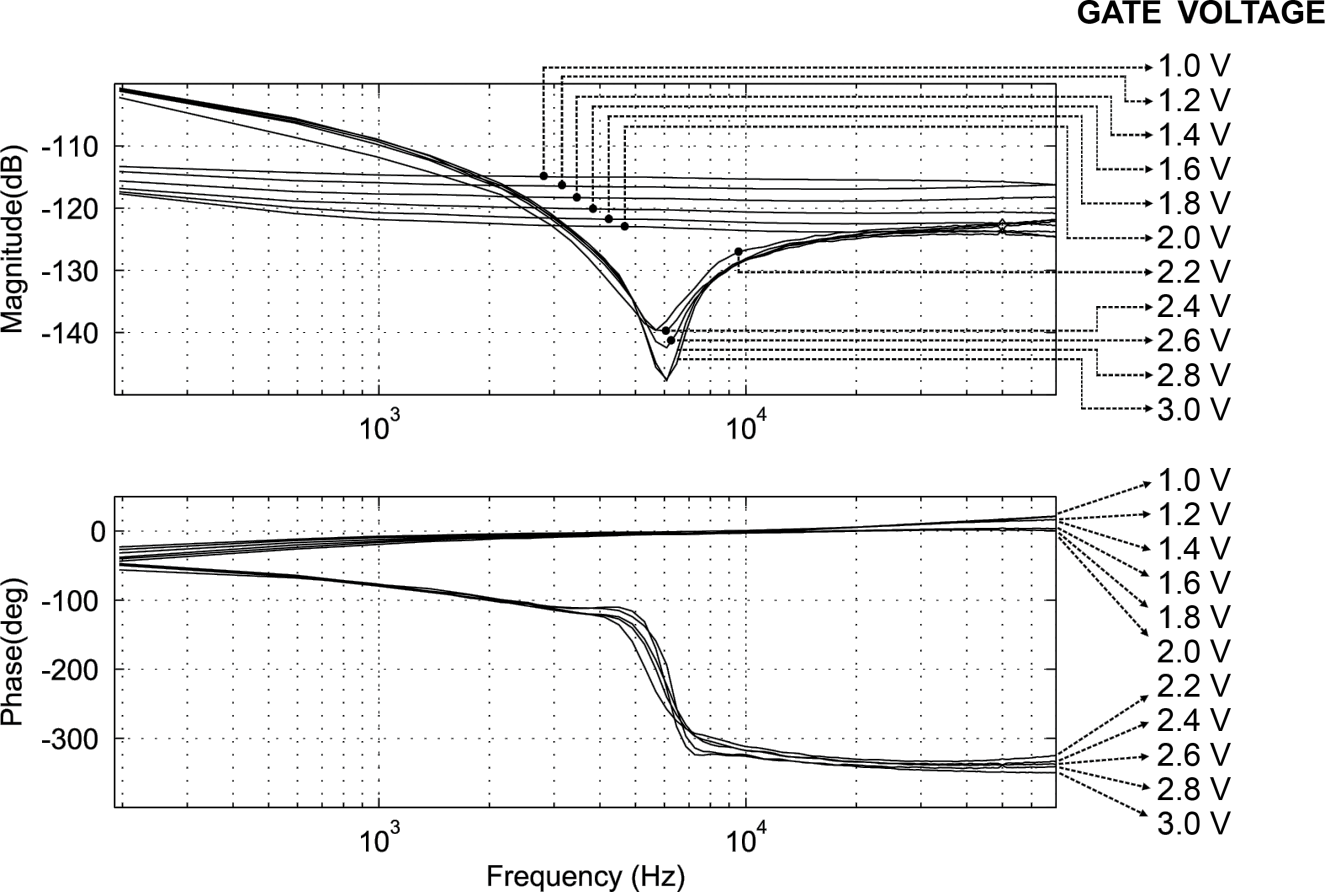
Admittance spectroscopy for gate voltages from 1.0 V to 3.0 V.

**Figure 12:**
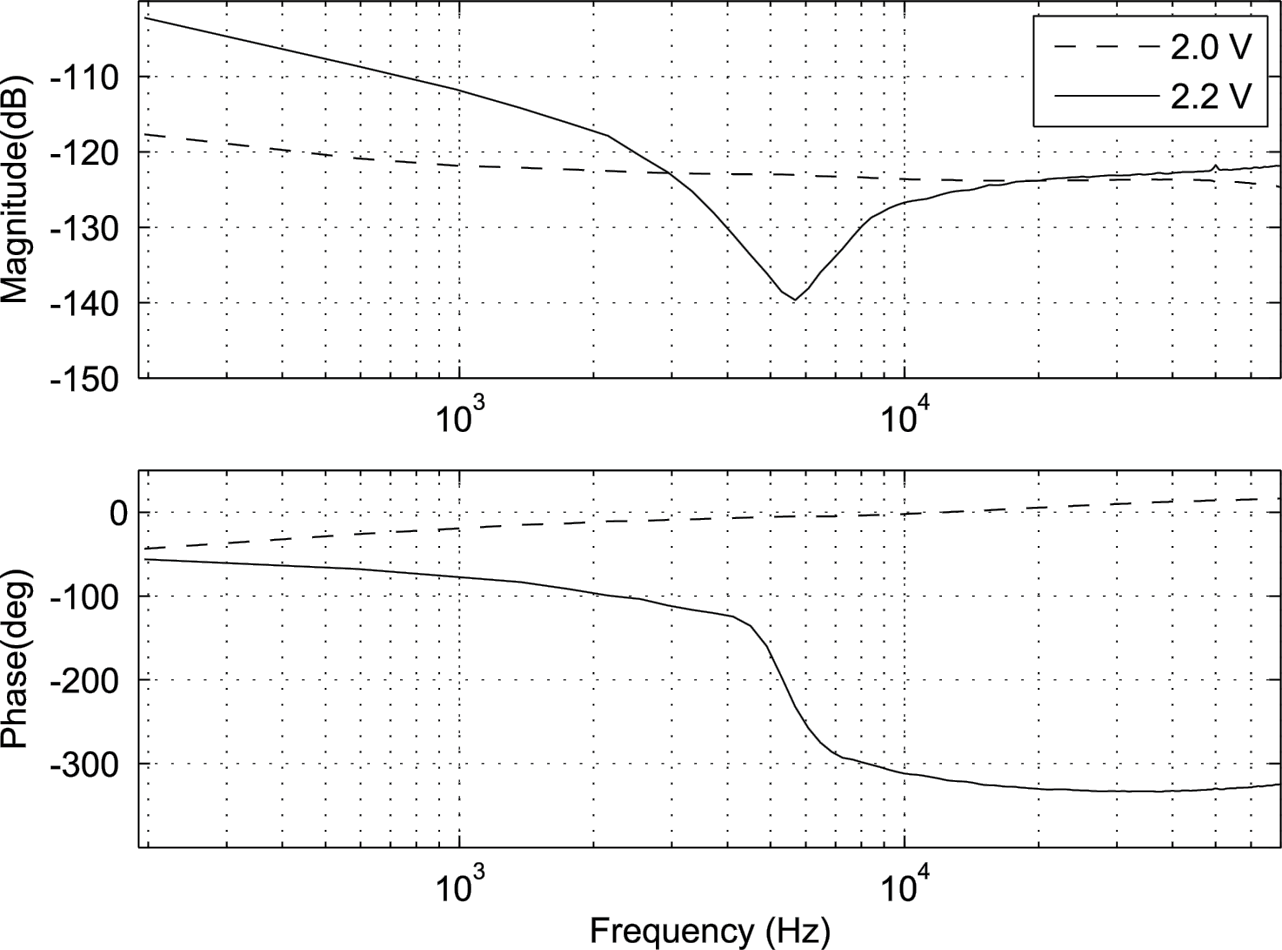
Admittance spectroscopy for gate voltages of 2.0 V and 2.2 V.

Figure 11 shows the computed frequency responses (admittance spectroscopy) when the gate voltage was changed from 1.0 V to 3.0 V. The figure shows an almost linear drop in the gain curve when the gate voltage is below 2.0 V. When the gate voltage drops from 2.5 V to below 2.0 V, *I*_ds_ drops quickly because it reaches the threshold voltage (2.0 V) that turns the channel to an off-state (while it was on-state at 2.5 V). Figure 12 shows the admittance spectroscopies for the gate voltages around the threshold value where the dynamics change rapidly.

## Conclusion

Silicon nanowire-based field-effect transistors (SiNW FETs) have been experimentally used for direct, label-free, highly selective, real-time detection of biological and chemical targets at very low concentrations. Most SiNW FET detectors are fabricated with SOI wafers, in which the back-gate contact is used to control the conductivity of the SiNW with the box layer. The molecules (detection targets) vary the surface potential of the transistor surface oxide. The leakage currents in those oxide layers affect the current between the source and drain. Recent studies indicate that the leakage current is frequency dependent. Measuring such characteristics can provide valuable tools to validate the functionality of the used transistors, and possibly have advantages in developing new frequency-domain-based detection technologies utilizing SiNW FETs.

This paper has presented fast frequency-domain methods with which to measure and characterize the leakage current. The inverse-repeat binary sequence (IRS) was applied, and the characterizing frequency responses were measured through Fourier methods. The experimental results showed that the leakage current strongly changes its frequency-domain properties at a certain gate voltage. This voltage value corresponds to the threshold voltage of the device.

The presented methods can be implemented cost-effectively, and provide responses within a few seconds. The applied techniques can also be used for measuring frequency responses other than the leakage current. The methods can be applied, for example, in analyzing the impedance of the nanowire in SINW FETs. This will be one part of the future work of the authors. Other future work will include further analysis of the leakage current, studying alternative excitation signals, implementing fully automated measurement systems, and designing new frequency-domain-based detection technologies.
